# Vertebral compression fractures in an HIV-positive patient with steroid-induced Cushing syndrome: a case report

**DOI:** 10.4076/1757-1626-2-7034

**Published:** 2009-06-24

**Authors:** David Leitman, Kenneth Ross

**Affiliations:** 1 Medical Education, A.T. Still University-Kirksville College of Osteopathic Medicine800 W. Jefferson Street, Kirksville, MO 63501USA; 2 Graduate Medical Education, Des Peres Hospital2345 Dougherty Ferry Rd, St Louis, MO 63122USA

## Abstract

The following case describes the presentation, diagnosis, and treatment of a 49 year-old patient with back pain in the thoracic spine and lower extremity edema. His initial presentation, coupled with a history of trauma, required an extensive work-up to determine the source of the back pain and edema. The patient was determined to have two thoracic vertebral compression fractures, bilateral lower extremity edema of unknown origin, and osteoporosis. After diagnosis, several specialists were consulted to determine the cause of the patient’s osteoporosis. Treatment consisted of kyphoplasty, physical therapy, and medication to prevent the progression of osteoporosis.

## Introduction

Compression fractures are most commonly seen in elderly females secondary to osteoporosis [1,2]. Due to their increased risk of developing fractures, this patient population is well screened for osteoporosis [2]. Unfortunately, younger patients, especially young men, may not be diagnosed with osteoporosis, even when they have multiple risk factors for developing the disease. Currently, the literature indicates that males between 50 and 69 years-old should have a DEXA scan if they have multiple risk factors for developing osteoporosis [2]. This guideline fails to identify those individuals under age 50, at high risk of developing osteoporosis. This case presentation describes a male with multiple risk factors who failed to be screened for osteoporosis.

## Case presentation

A 49 year-old Caucasian male presented with progressively worsening pain in the thoracic spine for 10 days and painful bilateral lower extremity edema. The patient had a history of lower thoracic and lumbar vertebral fractures, which resulted from a traumatic fall down a flight of stairs three months prior to this hospitalization. Those vertebral fractures were treated with six serial kyphoplasty procedures, with the final two vertebral fractures having been treated three weeks prior to his current hospitalization.

On admission, the patient was found to have severe persistent asthma and HIV. Psychosocial history was positive for a twelve-year history of IV drug abuse and a twenty-year history of alcohol dependence. He stopped drinking alcohol and using IV drugs eleven years ago. A thorough review of systems revealed increased fatigue and weakness since his traumatic fall. He also noted that he began developing “stretch marks” on his abdomen one month prior to this admission. The remainder of the review of systems was unremarkable.

Physical exam revealed an afebrile, obese male grimacing in severe pain, with no signs of cyanosis. He was 175 cm tall, weighed 129 kg, and had a BMI of 42.1. Vitals were as follows: BP: 154/98; RR: 28; HR: 82; Temp: 97.7F; O2 Sat: 98% on room air. The patient was alert and oriented to person, place, and time. Diffuse bilateral wheezes were present on chest auscultation. Large diffuse striae covered the entire abdomen. Bilateral 2/4 lower extremity edema was present. Dorsalis pedis pulses were 1/4 bilaterally. Capillary refill was within the normal range at 1.5 seconds bilaterally. The remainder of the physical exam was unremarkable and within normal limits.

The initial differential diagnosis was quite broad and included the following diagnoses: lower extremity edema of unknown origin, thoracic disk herniation, osteomyelitis, vertebral compression fracture, lower extremity arterial stenosis, and deep vein thrombosis (DVT). In order to determine the source of his pain and edema, laboratory and imaging studies were performed. Due to the patient’s history of vertebral compression fractures and disk herniation, an MRI of the thoracic spine was ordered. The MRI revealed new compression fractures at T6 and T7, with no changes in disk appearance from the MRI taken three months prior. Arterial stenosis was suspected because the patient had decreased dorsalis pedis pulses bilaterally. A bilateral lower extremity multilevel arterial Doppler ultrasound displayed normal triphasic waveforms throughout both lower extremities, and the Ankle Brachial Index was 1.0 at the distal point in both the right and left lower extremity. A bilateral venous Doppler was negative for DVT. A dual energy x-ray absorptiometry A DEXA scan was ordered to screen for osteoporosis. The patient had a bone mineral density (BMD) of -2.8. BMD that is 2.5 standard deviations below the mean (T-score -2.5) is considered osteoporosis [1].

Laboratory evaluation revealed a hemoglobin of 12.5 g/dl and a hematocrit of 37%. The white blood cell (WBC) count was 10.2 × 10^3^/mm^3^ and the CD4+ count was 312 cells/ml. ACTH and cortisol levels were ordered because Cushing syndrome was suspected given the patient’s body habitus and the presence of abdominal striae. The ACTH level was 2.1 (normal: 9-52 pg/ml) and the cortisol level was 3.7 µg/dl (normal: 5-24). An ACTH stimulation test resulted in a final cortisol level of 19.1 µg/dl, which indicated Cushing syndrome was secondary to exogenous steroid use. Given the patient’s history of HIV and osteoporosis, an androgen profile was also order to determine if the patient had hypogonadism. The following results were obtained: FSH: 6.2 IU/L (normal: 1.5-12.4 IU/L), LH: 8.9 IU/L (normal: 7.0-24.2 IU/L), total testosterone 271 ng/ml (normal: 350-890 ng/ml) and free testosterone: 37 pg/ml (normal: 44-244 pg/ml).

The imaging determined that the patient had vertebral compression fractures at T6-T7 and ruled out a new thoracic disk herniation and osteomyelitis as the source of pain. The laboratory studies, coupled with clinical examinations determined that that patient had steroid-induced Cushing syndrome, hypogonadism, and osteoporosis. Treatment consisted of kyphoplasty of T6-T7 to improve the vertebral defect, physical therapy to decrease the patient’s lower extremity edema, and medication changes to treat his hypogonadism, osteoporosis and steroid-induced Cushing syndrome. The patient was prescribed 10 g daily of testosterone topical (1%) to treat the hypogonadism. Calcium 1200 mg/day, ergocalciferol 50,000 units weekly, and alendronate 70 mg weekly were used to treat the osteoporosis. The patient was changed from fluticasone/salmeterol 500/50 one puff BID to 100/50 one puff BID and was started on theophylline 400 mg/day. Following these treatment modalities, the patient was able to increase his ambulation, which resulted in decreased leg edema and pain. At the time of discharge, the patient had minimal thoracic spine pain and trace lower extremity edema.

## Discussion

This case demonstrates how HIV, steroid-induced Cushing syndrome, and hypogonadism can combine to cause osteoporosis in a middle-aged male. The case also displays how severe osteoporosis can lead to a disastrous outcome following trauma. This discussion will address the risk factors leading to the development of osteoporosis in our patient, as well as the modalities used to treat the patient.

Glucocorticoids are the leading cause of medication-induced osteoporosis [3]. Fracture risk increases within three months of starting oral or high-dose inhaled glucocorticoids [3]. Glucocorticoids increase bone loss by multiple mechanisms including: inhibition of osteoblast function, stimulation of bone resorption, increase in urinary calcium loss, decrease in calcium absorption, and reduction in adrenal and testicular androgen secretion. Bisphosphonates are the cornerstone of treatment for steroid-induced osteoporosis [3]. Decreasing the amount of inhaled fluticasone and adding alendronate were two methods used to treat this patient’s osteoporosis and these modalities are the favored modalities in the literature.

Due to the severity of the patient’s osteoporosis, 20 mcg of teriparatide daily for six months to two years could have been used initially, instead of alendronate. Teriparatide has been shown to have a greater efficacy in preventing vertebral fractures than alendrote and other bisphosphonates [4]. If teriparatide was used, the bisphosphonate should have been discontinued. The literature indicates that there is no added benefit to taking a bisphosphonate with teriparatide [4].

There is a controversy as to whether highly active anti-retroviral therapy (HAART) is responsible for decreased bone density in HIV patients or if the disease itself is a risk factor for osteoporosis. A recent study indicates that patients taking HAART therapy are at increased risk of developing osteopenia and osteoporosis due to the adverse effects protease inhibitors (PIs) have on conversion of (25)-OH vitamin D to (1,25)-OH vitamin D. An in vitro study demonstrated that ritonavir, indinavir, and nelfinavir decreased conversion to active vitamin D by 80%, 66%, and 31%, respectively [5]. Another group found that although HIV-positive patients tend to have low BMD, the BMD is independent of the treatment type. In the study, one third was treated with PIs, one third was treated without PIs, and one third elected to have no treatment. The study showed no significant difference between the three groups with regards to BMD [6].

There are only a few studies that have evaluated whether switching HAART can be beneficial. One study evaluated 18 patients who switched from a protease inhibitor-based regimen to a non-nucleoside reverse transcriptase inhibitor-based regimen. No improvements in BMD were observed on DEXA scanning after 48 weeks of follow-up in these patients [7]. Changing HAART is currently not recommended because no studies have shown that changing therapy will improve BMD [7]. As a result, our patient was continued on his HAART regiment after he was diagnosed with osteoporosis.

Men infected with HIV are more likely to be hypogonadal, as judged by generally lower serum testosterone concentrations, than men without HIV [8]. Hypogonadism is the best-characterized risk factor for osteoporosis in men. Although the cause of hypogonadism in HIV-infected males remains unclear, the presence of hypogonadism significantly increases the risk of osteoporosis in these patients. Providing supplemental testosterone to hypogonadal patients has been shown to decrease the number of osteoporotic fractures when used as an adjunct to bisphosphonates [9].

Kyphoplasty was used to repair the patient’s compression fractures. Studies have shown that kyphoplasty is an effective means to increase vertebral height and decrease kyphosis following vertebral compression fractures. Kyphoplasty was used instead of vertebroplasty because kyphoplasty completely restores vertebral body height in a greater percentage of patients than vertebroplasty [10].

A post-operative adverse effect of kyphoplasty and vertebroplasty is the development of new vertebral fractures [11,12]. Two retrospective studies found that patients treated with vertebroplasty had an increased rate of new vertebral fractures [11,12]. In a study with 432 patients treated with vertebroplasty, 84 patients had new vertebral fractures occurring within four months of the procedure [11]. In another study composed of 177 patients, 22 patients developed at least one vertebral fracture following vertebroplasty and 67 percent of the fractures involved adjacent vertebrae [12].

The cause of our patient’s new vertebral fractures was likely multifactorial. Both osteoporosis and alteration of mechanical forces on adjacent vertebrae secondary to kyphoplasty led to the development of compression fractures at T6 and T7. Since the patient had a history of severe persistent asthma, it was necessary to perform kyphoplasty in order to prevent the development of a severe kyphosis and a subsequent restrictive lung disease.

## Conclusion

This case demonstrates that gender and age cannot be the only variables used to determine if a patient warrants screening for osteoporosis. Our patient had not been screened for osteoporosis in the past because he was male and less than 50 years of age. He should have been screened because he had multiple risk factors for developing osteoporosis. A lack of screening, coupled with his risk factors and a traumatic fall resulted in severe vertebral injury, which continued to plague this patient for well over three months. Our patient illustrates the importance of screening patients with multiple risk factors for osteoporosis, especially those with a history of severe persistent asthma, HIV, and hypogonadism.

## List of abbreviations

ACTH: adrenocorticotropic hormone
BMD: bone mineral density
BMI: bone mineral density
BP: blood pressure
DEXA: dual energy x-ray absorptiometry
DVT: deep vein thrombosis
FSH: follicle stimulating hormone
HAART: highly active anti-retroviral therapy
HIV: human immunodeficiency virus
HR: heart rate
IV: intravenous
LH: luteinizing hormone
PI: protease inhibitor
RR: respiratory rate

## References

[bib-001] Lieberman S, Fishman MC, Klausner RD, Hoffman AR (2004). Diseases of the Parathyroid Gland and Bone. Medicine.

[bib-002] Lindsay R, Cosman F, Fauci AS (2008). Osteoporosis. Harrison’s Principles of Internal Medicine.

[bib-003] Quddusi S, Browne P, Toivola B, Hirsch IB (1998). Cushing syndrome due to surreptitious glucocorticoid administration. Arch Intern Med.

[bib-004] Saag KG, Shane E, Boonen S, Marín F, Donley DW, Taylor KA, Dalsky GP, Marcus R (2007). Teriparatide or alendronate in glucocorticoid-induced osteoporosis. N Engl J Med.

[bib-005] Tebas P, Powderly WG, Claxton S, Marin D, Tantisiriwat W, Teitelbaum SL, Yarasheski KE (2000). Accelerated bone mineral loss in HIV-infected patients receiving potent antiretroviral therapy. AIDS.

[bib-006] Amiel C, Ostertag A, Slama L, Baudoin C, N’Guyen T, Lajeunie E, Neit-Ngeilh L, Rozenbaum W, De Vernejoul MC (2004). BMD is reduced in HIV-infected men irrespective of ART treatment. J Bone Miner Res.

[bib-007] Tebas P, Yarasheski K, Henry K, Claxton S, Kane E, Bordenave B, Klebert M, Powderly WG (2004). Evaluation of the virological and metabolic effects of switching protease inhibitor combination antiretroviral therapy to nevirapine based therapy for the treatment of HIV infection. Res Human Retroviruses.

[bib-008] Klein RS, Lo Y, Santoro N, Dobs AS (2005). Androgen levels in older men who have or who are at risk of acquiring HIV infection. Clin Infect Dis.

[bib-009] Poretsky L, Can S, Zumoff B (1995). Testicular dysfunction in human immunodeficiency virus-infected men. Metabolism.

[bib-010] Hiwatashi K, Sidhu R, Lee RK (2005). Kyphoplasty versus vertebroplasty to increase vertebral body height: a cadaveric study. Radiology.

[bib-011] Trout AT, Kallmes DF, Kaufmann TJ (2006). New fractures after vertebroplasty: adjacent fractures occur significantly sooner. Am J Neuroradiol.

[bib-012] Uppin AA, Hirsch JA, Centenera LV (2003). Occurrence of new vertebral body fracture after percutaneous vertebroplasty in patients with osteoporosis. Radiology.

